# Improving Risk Stratification of Patients With Chest Pain in the Emergency Department

**DOI:** 10.7759/cureus.33202

**Published:** 2023-01-01

**Authors:** Yusuf Altunoz, Banu Karakus Yilmaz, Hatice Topcu, Gökhan Cetinkal, İbrahim İkizceli, Yavuz Yigit

**Affiliations:** 1 Emergency Medicine Specialist, Şanlıurfa Viranşehir Devlet Hastanesi, Şanlıurfa, TUR; 2 Emergency Department, Alanya Alaaddin Keykubat University, Medical School, Antalya, TUR; 3 Emergency Medicine, Sisli Hamidiye Etfal Research and Training Hospital, Istanbul, TUR; 4 Cardiology, Sisli Hamidiye Etfal Research and Training Hospital, Istanbul, TUR; 5 Emergency Department, Istanbul University School of Medicine, Istanbul, TUR; 6 Emergency Medicine, University of Health Sciences, Kocaeli Derince Training and Research Hospital, Kocaeli, TUR

**Keywords:** emergency medi, early identification and diagnosis, hearts3 score, : acute coronary syndrome, anginal chest pain

## Abstract

Objective: The HEARTS3 score is used to predict acute coronary syndrome by evaluating the findings of chest pain patients at the end of the second hour. Additionally, the American College of Cardiology (ACC)/American Heart Association (AHA) 2014 non-ST elevation acute coronary syndrome (NSTE-ACS) management guideline suggests assessing cardiac troponin levels at the third and sixth hours as a class 1A recommendation. This study aimed to explore the value of the HEARTS3 score for the evaluation of patients with chest pain and its utility for determining whether a patient is eligible for early discharge from the emergency department.

Material and methods: This study was prospectively conducted between March 1, 2016 to May 31, 2016 at the ED of the Research and Training Hospital in İstanbul. A total of 136 patients were evaluated, and HEARTS3 scores were calculated at the second, third, and sixth hours. Receiver operating characteristic (ROC) curves were used to calculate the specificity, sensitivity, negative predictive value (NPV) and positive predictive value (PPV) of these scores. The primary outcome was the occurrence of major adverse cardiac events (MACEs) within 30 days.

Results: In total, 29 patients with MACEs and 107 patients without MACEs were identified within 30 days. Based on the ROC curve, the cutoff value for early discharge was 6. The area under curve (AUC) values were 0.943, 0.963 and 0.976 at the second, third, and sixth hours, respectively. The sensitivity of the second-hour HEARTS3 score was 96.6%, and the NPV was 98.6%. Both the sensitivity and NPV reached 100% at the sixth hour.

Conclusion: The HEARTS3 score was considered a feasible method for the prediction of MACEs. We concluded that a patient with a HEARTS3 score less than 6 may be discharged without serial troponin and ECG examination.

## Introduction

One of the most frequent complaints is chest pain in emergency departments (EDs). However, this symptom may be observed in many diseases that are not typically treated in the ED and may be a symptom of a serious cardiac or noncardiac disease [1]. The management guideline for non-ST elevation acute coronary syndrome (NSTE-ACS) presented by the American College of Cardiology was updated in 2014. The associations have proposed to use risk categorization models [2]. Same guidelines reported that the Sanchis score, Vancouver rule, Hess prediction rule, HEART score, and HEARTS3 score can be used for the management of unidentified chest pain [3-7].

The HEART score is used to classify patients as low, middle, and high risk based on chest pain, presence of cardiac risk factors, age, arrival electrocardiography (ECG) findings and cardiac troponin enzyme values [5]. In a study performed in 2010, HEART, GRACE and TIMI scores were used to determine the risk of myocardial infarction (MI) or acute coronary syndrome (ACS) survival within six weeks by evaluating patients who visited the clinic with chest pain, and the HEART score was found to be superior to GRACE and TIMI scores [7]. Another study reported that the HEARTS3 score was superior to the HEART, HEART (weighted) scores in prognosticating four-week MI and ACS risk among patients with chest pain [6].

Even though the findings at the end of the second hour are evaluated in the HEARTS3 score, the American College of Cardiology (ACC)/American Heart Association (AHA) 2014 NSTE-ACS management guideline suggests assessing cardiac troponin levels at the third and sixth hours from the onset of pain in all patients with suspected cardiac chest pain as a class 1A recommendation.

This study aimed to explore the value of HEARTS3 score for the evaluation of patients with chest pain and its utility for the assessment of early discharge from the ED.

## Materials and methods

Study design and center

This is a cross-sectional single-center study. The study was prospectively administered between March 1, 2016 and May 31, 2016 at the ED of a tertiary care setting with approximately 300,000 emergency admissions per year. The local ethics committee approved (#615) the study and then all the patients who were enrolled in this study were informed with a consent form. This study was administered according to the Declaration of Helsinki principles.

Study population

**Figure 1:**
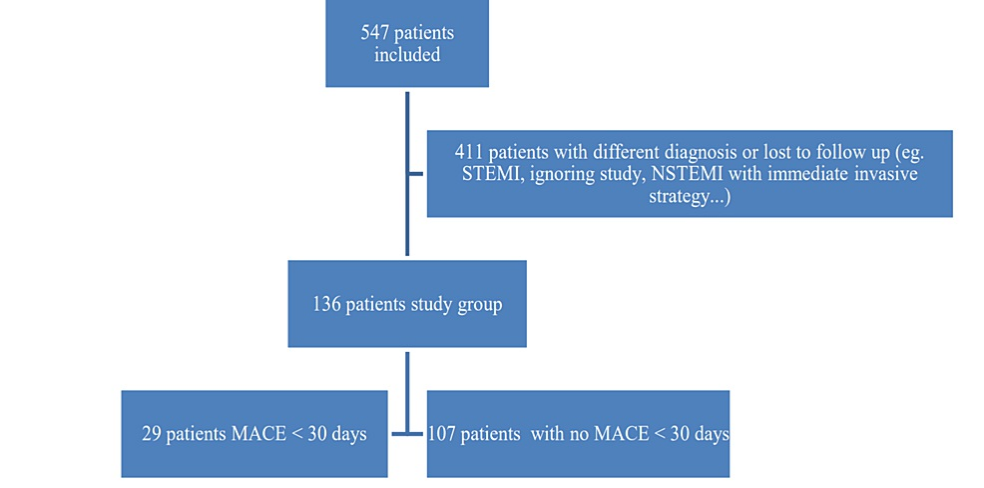
Flow chart MACE: major adverse cardiac events, NSTEMI: non-ST-elevation myocardial infarction, STEMI: ST-elevation myocardial infarction

Patients accepted to the ED with chest pain complaint and suspected to have ACS were screened for eligibility. Patients with HEARTS3 scores calculated at the second, third, and sixth hours and those with information about MACEs within 30-day period. Patients older than 18 years of age were included in this study. Patients who have trauma-related chest pain; unconscious patients (e.g., patients who were accepted to the ED because of cardiac arrest or intubated); patients without cardiac enzyme and ECG follow-up data; patients with chest pain who received a definitive diagnosis (STEMI, pericarditis-myocarditis, pulmonary embolism, pneumothorax, aortic dissection, or intra-abdominal cause of chest pain, such as peptic ulcer-cholecystitis-pancreatitis, etc.); patients in the intensive care unit with multiple organ dysfunction and pneumonia; patients who refused follow-up; patients who could not be followed after a 30-day period; and patients who were refused to join the study were excluded (Figure 1).

Study protocol

In the study, the HEARTS3 score was calculated by evaluating patients' characteristics of chest pain, age, coronary risk factors, sex, ECG, with cardiac troponin levels at admission time together with at the second hour. For the same patients, ECG and cardiac troponin enzyme levels were obtained at the third and sixth hours, and the same score was calculated again using data obtained at these time points. Patients' chest pain complaints were divided into three groups: high suspicious pain, noncardiac pain, and possible suspicion of cardiac pain. Coronary risk factors include the following: age >45 years; active or >10 years smoking history; male sex; coronary artery disease (CAD) history; and being overweight (BMI > 30 kg/m2), diabetes, hypertension, and dyslipidemia. The CAD story was based on past MI, percutaneous coronary intervention (PCI), coronary artery bypass grafting (CABG), sudden cardiac death, or unknown cause of death. ECG evaluations were made with the emergency physician who was primarily responsible for the patient. Patients were divided into three groups according to ECG findings (Minnesota criteria were used to define patients): normal ECG, abnormal ECG which has ischemic ST segment depression with abnormal ECG changes without ischemic ST segment depression [8]. Serial ECGs taken at the second, third, and sixth hours were similarly evaluated by the emergency physician, and patients were divided into three groups: no changes, diagnostic ischemic changes, and nondiagnostic changes.

Conventional troponin I (TnI) was studied as a cardiac biomarker. At least 4 ml of a venous blood sample was obtained from the patient each time and given to the emergency biochemistry laboratory to assess cardiac TnI levels. The blood sample was centrifuged for 10 minutes at least at 4000 rpm and then assessed in a COBAS e601 (Roche, Basel, Switzerland) series device. The threshold value for that device was -0.600 ng/mL. Echocardiography was performed by a cardiologist for all patients.

Outcome measures

At the end of the 30-day period, the presence of any myocardial infarction, PCI or CABG administration, fatal arrhythmias, and sudden cardiac death or sudden death with unknown etiology were defined as MACEs. MI was defined as 'the rise or drop of cardiac biomarker values and one of the following: ischemic symptoms; new or presumed significant ST-T changes or new left bundle branch block; pathological Q waves in ECG; imaging of new myocardial tissue loss; and recognition of an intracoronary thrombus while angiography or autopsy' according to ACC and European Society of Cardiology (ESC) criteria, which was updated in 2012 [8]. Ventricular fibrillation and grade 3 atrioventricular (AV) block were assessed in life-threatening arrhythmias.

Data analysis

The descriptive statistics are presented as frequency distribution, the mean, (±) standard deviation (SD) and percentage. In addition, the other statistical methods were used as descriptive statistics, Pearson chi-square test, the Mann Whitney u test, receiver operating characteristic (ROC) curve, and Fisher's exact test. We used Chi-Squared Test with Yates Continuity Correction to analyse some HEARTS3 components (age, risk factors, troponin I values, serial ECG in two, three, and six hours) and emergency service diagnosis. The data were assessed using the SPSS v. 15 (IBM Corp., Armonk, NY, USA). p< 0.05 was accepted as the statistical significance level.

## Results

In this study duration, 70,559 patients were received in our emergency department. A total of 547 of these patients complained of chest pain and were evaluated in our study. The 136 patients who did not meet exclusion criteria and who obtained information about MACEs within a 30-day period were included in this study.

The patients’ mean age was 57.38 ± 11.23 years. Forty-five (33.1%) were female, and 91 (66.9%) of the patients were male. Relationship between the risk factors for the patients and MACE (+) and MACE (-) patients is provided in Table 1.

**Table 1:** Demographic characteristics of patients and distribution of CAD risk factors ^& ^: analyzed with Mann Whitney U, ^a^: analyzed with Fishers exact test, *:p < 0.05 CAD: Coronary artery disease, MACE: Major adverse cardiac events

CAD risk factors	MACE (+) (n=29) n(%)	MACE (-) (n=107) n(%)	p
Age^&^	67.10±11.26	54.75±14.26	0.003*
Smoke^a^	25 (86.2)	51 (47.7)	0.001*
Diabetes^a^	15 (51.7)	28 (26.2)	0.009*
Male Gender^a^	18 (62.1)	73 (68.2)	0.532
Hypertension^a^	18 (62.1)	37 (34.6)	0.007*
Dyslipidemia^a^	14 (48.3)	8 (7.5)	0.001*
CAD History^a^	19 (65.5)	37 (34.6)	0.003*
Family History^a^	9 (31)	30 (28.0)	0.752
Obesity^a^	6 (20.7)	10 (9.3)	0.093

**Figure 2:**
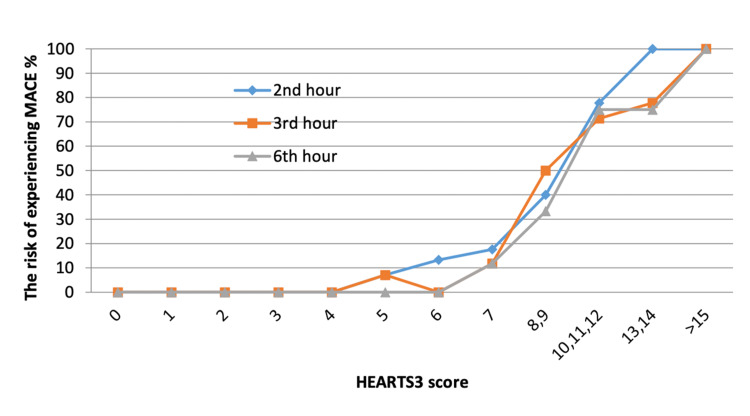
MACE ratios of scores calculated at the second, third, and sixth hours. MACE: major adverse cardiac events

The mean HEARTS3 score for all patients was 5.78 ± 3.78 for the second hour, 6.25 ± 4.39 for the third hour, and 6.46 ± 4.53 for the sixth hour in this study. The mean HEARTS3 scores of the MACE (-) and MACE (+) patients are provided in Table 2. Figure 2 shows the MACE ratios of scores calculated at the second, third, and sixth hours.

**Table 2:** HEARTS3 scores calculated at second, third, and sixth hours and AUC values AUC: area under curve, MACE: major adverse cardiac events

	2nd hour HEARTS3score	3rd hour HEARTS3 score	6th hour HEARTS3 score
Mean value in MACE (+) patients	8.83±1.58	9.52±1.55	9.79±1.29
Mean value in MACE (-) patients	4.32±2.19	4.42±2.30	4.52±2.34
Sensitivity (%)	96.6	96.6	100
Specificity(%)	67.3	64.5	62.6
Positive predictive value(%)	44.4	42.4	42
Negative predictive value(%)	98.6	98.6	100
AUC values	0.943	0.963	0.976

**Figure 3:**
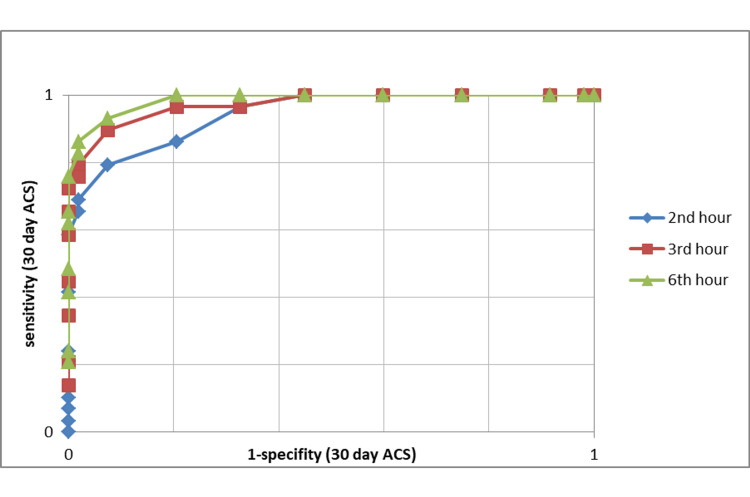
The HEARTS3 scores calculated at the second, third, and sixth hours to 30-day MACE with ROC curves AUC values:second hour - 0.943, third hour - 0.963, sixth hour - 0.976 MACE: major adverse cardiac events, AUC: area under curve, ROC: receiver operating characteristic, ACS: acute coronary syndrome

The HEARTS3 scores calculated at the second, third, and sixth hours to 30-day MACE were used to compare with ROC curves and are shown in Figure 3. Using the ROC curve, the cutoff value for early discharge values ​​was calculated as 6. The specificity, sensitivity, negative predictive value (NPV) and positive predictive value (PPV) of the HEARTS3 score were calculated based on this value. The 30-day MACE estimation rates are given in Table 2.

We noted that male sex did not show a statistically significant effect on MACE survival in our study (p=0.532). The risk factors for coronary artery disease with the HEARTS3 score components of the patients are individually compared with MACE (-) and MACE (+) patients (Table 3 and Table 4).

**Table 3:** Distribution of HEARTS3 score components on patients ^&^: analyzed with Pearson chi square test, ^a^: analyzed with Chi-Square Test with Yates Continuity Correction, *:p <0.05 CP: Chest pain, MACE: Major adverse cardiac events

Variable	HEARTS3 score points	MACE (+) patients (n=29), n (%)	MACE (-) patients (n=107), n (%)	p
History^&^				
Non-cardiac CP	0	0 (0)	40 (37.4)	0.001*
Possible cardiac CP	1	6 (20.7)	48 (44.8)	
Probable cardiac CP	4	23 (79.3)	19 (17.8)	
ECG (baseline)^&^
Normal	0	8 (27.6)	52 (48.6)	0.001*
Non-spesific ST changes	1	8 (27.6)	42 (39.3)	
İschemic ST depression	3	16 (44.8)	13 (12.1)	
Age^a^				
<45	0	0 (0)	27 (25.2)	0.030*
>45	1	29 (100)	80 (74.8)	
Risk factors^a^				
<3	0	1 (3.4)	34 (31.8)	0.002*
≥3	1	28 (96.6)	73 (68.2)	
Troponin I (baseline)^a^				
<0.6 ng/dl	0	23 (79.3)	107 (100)	
0.6 – 1.8 ng/dl	2	3 (10.3)	0 (0)	0.001*
>1.8 ng/dl	5	3 (10.3)	0 (0)	
Sex^&^				
Female	0	11 (37.9)	34 (31.8)	0.532
Male	1	18 (62.1)	73 (68.2)	
Serial ECG (2. hour)^a^				
No change	0	20 (69.0)	104 (97.2)	0.001*
non-diagnostic change	2	4 (13.8)	3 (2.8)	
diagnostic change	5	5 (17.2)	0 (0)	
Serial troponın I (2. hour)^a^				
<0.1 ng/dl change	0	15 (51.7)	104 (97.2)	
0.1-0.3 ng/dl change	2	6 (20.7)	2 (1.9)	0.001*
>0.3 ng/dl change	5	8 (27.6)	1 (0.9)	

**Table 4:** Adding third and sixth hour variable ^a^:analyzed with Chi-Square Test with Yates Continuity Correction, *:p <0.05 MACE: Major adverse cardiac events

Variable	HEARTS3 score points	MACE (+) patients	MACE (-) patients	p
		(n=29), n(%)	(n=107), n(%)	
Serial ECG (3. hour)^a^				
No change	0	17 (58.6)	101 (94.4)	0.001*
non-diagnostic change	2	4 (13.8)	4 (3.7)	
diagnostic change	5	8 (27.6)	2 (1.9)	
Serial troponın I (3. hour)^a^					
<0.1 ng/dl change	0	10 (34.5)	103 (96.3)	0.001*
0.1-0.3 ng/dl change	2	5 (17.2)	1 (0.9)	
>0.3 ng/dl change	5	14 (48.3)	3 (2.8)	
Serial ECG (6. hour)^a^
No change	0	16 (55.2)	100 (93.5)	0.001*
non-diagnostic change	2	4 (13.8)	4 (3.7)	
diagnostic change	5	9 (31.0)	3 (2.8)	
Serial troponın I (6. hour)^a^					
<0.1 ng/dl change	0	10 (34.5)	102 (95.3)	0.001*
0.1-0.3 ng/dl change	2	1 (3.4)	1 (0.9)	
>0.3 ng/dl change	5	18 (62.1)	4 (3.7)	

Both the emergency service diagnosis and echocardiography findings were compared between the MACE (-) and MACE (+) patients. These factors were significantly different between these patients (P < 0.001, for both) (Table 5).

**Table 5:** Distribution of emergency service diagnosis and ECHO images over MACE ^&^: analyzed with Pearson chi square test, ^a^: anaylzed with Chi-Squared Test with Yates Continuity Correction, *:p < 0.05 MACE: Major adverse cardiac events, AP: Angina pectoris, USAP: Unstable angina pectoris, NSTEMI: non-ST-elevation myocardial infarction, EF: ejection fraction

	MACE (+)	MACE (-)	p
	n=29 (%)	n=107 (%)	
Emergency service diagnosis^a^			
Stabil AP	2 (6.9)	63 (58.9)	0.001*
USAP	7 (24.1)	40 (37.4)	
NSTEMI	20 (69.0)	4 (3.7)	
Echocardiography^&^
Normal	9 (31.0)	57 (53.2)	0.001*
Low EF, hypokinesia, akinesia	20 (69.0)	11 (10.3)	

PCI was applied to 18 (62.1%) of the MACE (+) patients. CABG was performed in nine (31.1%) patients. Life-threatening arrhythmia (ventricular fibrillation) was found in one (3.4%) patient and sudden cardiac death was found in one (3.4%) patient.

## Discussion

In this prospective study aiming to evaluate early discharge of the patients admitted to the ED with chest pain and to assess the efficacy of HEARTS3 score in predicting the development of MACEs, and we found that the HEARTS3 scores may predict the 30-day risk of MACEs at the second hour of ED admission and can help with the early discharge of chest pain patients.

A significant relationship was noted between HEART and HEARTS3 scores and the risk of MACE after four to six weeks of follow-up in previous studies. It has been shown that HEART and HEARTS3 scores provide reliable estimates for triage patients who have chest pain [6,9,10]. Additionally, easy and practical methods used for these calculations, many studies have found that HEART and HEARTS3 scores were more sensitive and specific than TIMI, PURSUIT, and GRACE scoring systems [6]. The HEARTS3 score, which includes the ECG findings and cardiac troponin values ​​of the patients at the second hour, yields more reliable results than the HEART score.

A multicenter prospective study managed by Backus et al. in 2013 evaluated 2388 patients, and the HEART score ability to predict the risk of MACEs after six weeks was investigated. They reported that the MACE rate was 1.7% when the HEART score was 0-3, 16.6% among those with a score of 4-6, and 50.1% among those with a score of 7-10 [11]. The study managed by Six et al. evaluated 122 patients who suffered from chest pain, and the relation between the risk of three-month MACE survival and the HEART score was assessed [9]. The MACE rate was respectively 2.5%, 20.3% and 72.7% in patients with a HEART score of 0-3; 4-6; and 7-10 [9]. The multicenter another study managed by Six et al. in 2013, 2906 patients were retrospectively assessed, and the 30-day MACEs prediction power of the HEART score was reported to be more reliable than that of the TIMI risk score (C statistics: 0.83 to 0.75) [12]. In light of these three studies, it was advocated that patients that have 0-3 point HEART scores do not require examination and can be discharged. Patients with 4-6 points require further examination and treatment, and patients with 7-10 points may require early invasive procedures [9,11,12].

In 2012, in a study conducted by Francis et al., 2148 patients with chest pain were retrospectively assessed, and the predictive power of HEART, HEART (weighted), HEARTS3 scores on 30-day MACEs survival were compared. Using the ROC curves of these scores, it was reported that the HEARTS3 score is better than the HEART score for MACE estimating power [6]. In our study, 136 patients were evaluated prospectively, and the second-hour HEARTS3 score successfully estimated the 30-day MACEs rate with a sensitivity of 96.6% and a negative predictive value of 98.6%. Our results are compatible with the current literature.

In 2013, Backus et al. compared all components of the HEART score between MACEs-positive and MACEs-negative patient groups, and all components (chest pain, age, risk factors, ECG, and troponin) were considered independent determinants of MACEs [11]. Despite the presence of chest pain, ECG and troponin were independent determinants of these components in a study managed by Six et al. in 2008, and no significant statistical significance was found for age (p=0.2847) or cardiac risk factors (0.0827) [9]. Furthermore, the study managed by Six et al. in 2013, each of the cardiac risk factors and MACE-positive and MACE-negative groups were compared separately, and the risks were independently determined for male sex, diabetes mellitus (DM), hypercholesterolemia, HT, CAD, family history, MI history, and CABG history [12]. Francis et al. did not provide such data [6]. In our study, each component of the HEARTS3 scores was compared between the MACE-positive and MACE-negative patient groups. The history of chest pain, risk factors about cardiac events, age, significant ECG changes, and significant troponin changes were identified as independent determinants, but male sex was not (p=0.532).

Each of the cardiac risk factors was compared separately between MACE-positive and -negative groups, and smoking, DM, CAD history, dyslipidemia and age were identified as independent risk factors for MACEs. No significant differences in obesity (p=0.093), family history (p=0.752) and male sex (p=0.532) were noted. In our study, the relationship between the components of HEARTS3 score and the cardiac risk factors associated with MACEs is an expected result, as reported in the literature. The inability to establish a meaningful relationship between male sex and MACEs was attributed to the fact that the female patients in our study were older than males. On the other hand, we think that the lack of meaningful findings of obesity and family history is related to the distribution of the patient population in our study.

Noninvasive imaging modalities applied to the emergency department and patients' emergency services diagnosis have not been previously studied. The transthoracic echocardiography findings of the patients in our study were examined separately in the MACE-positive and MACE-negative groups. There were 97 patients with echocardiography results, and 66 of these patients had normal results. MACEs were noted in nine (13.6%) of these patients. MACEs were observed in 20 (64.5%) of 31 patients with low ejection fraction (EF), hypokinesia or akinesia on echocardiography. Noninvasive imaging methods are effective in the management of chest pain patients.

Aydin et al. compared HEART and HEARTS3 scores of patients who developed MACE or not. They found the scores higher for MACE-positive patients [13]. Regarding the patients' ED diagnosis, 65 patients were discharged with a stable angina pectoris (STAP) diagnosis; 24 patients were diagnosed with NSTEMI, and 47 patients had unstable angina pectoris (USAP), so all of them were hospitalized in the coronary intensive care unit. MACEs occurred in two (3.1%) patients with STAP, seven (14.9%) with USAP, and 20 (83.3%) patients with NSTEMI. The statistical difference (p<0.001) was recorded between the MACE-positive and MACE-negative groups according to the last diagnosis of the patients in the emergency department. We think that the diagnoses received in ED are an important determinant of MACEs.

The relationship between the increase in HEART and HEARTS3 scores and the development of MACEs has been studied in previous studies [6,9-11]. In these studies, the HEART score was divided to three groups: poor (0 - 3 points), moderate (4 - 6 points) and elevated risk (7 - 10 points). However, a cutoff value was not used for the HEARTS3 score. A significant cutoff value of 6 was calculated using the ROC curve for each of the three scoring assessments in our study. Our findings support that a low HEARTS3 score is a strong predictor for early discharge of patients with chest pain that have 96.6% sensitivity and 98.6% negative predictive value. We think that these values ​​do not make a meaningful difference even though they increase to 100% at the sixth hour. In the third hour, there was no change in these values.

Study limitations

The most important limitation of this study is the small number of study participants and that it is a single-center study.

In our study, the patients were evaluated by an emergency medical specialist in ED. Classification of chest pain characteristics of patients, interpretation of ECG and chest radiographs, and outcome diagnoses have led to differences in knowledge and experience among physicians.

Another important limitation of this study was that we had to use the conventional TnI, although newly published guidelines recommend using hs-TnI.

## Conclusions

Detailed assessment of chest pain characteristics and determination of cardiac risk factors will provide important benefits to the emergency physician. Additionally, the HEARTS3 score was considered a feasible method in the prediction of MACE as an easily calculated score. We concluded that patients with a HEARTS3 score less than 6 may be discharged without serial troponin and ECG examination. To assess the practical significance of this result, well-designed studies with larger patient series are needed.
